# Mixed Germ Cell Tumour in a 58-Year-Old Post-menopausal Woman: A Case Report

**DOI:** 10.7759/cureus.109076

**Published:** 2026-05-18

**Authors:** Victor C Okebalama, Ogonna Arisi, Dorothy Oragwa, Bonaventure M Ogbodo, Olutomiwa Omokore, Kenelumchukwu Ekezie, Obioma Aririsukwu, Eberechuckwu Onyemachi, Nwora Oli, Onyebuchukwu Eke, Cyprian Okoronkwo, Odutola Odugbemi, Joshua Odeyinka, Esosa Imafidon

**Affiliations:** 1 Histopathology, Babcock University Teaching Hospital, Ilishan-Remo, NGA; 2 Emergency Medicine, University Hospital Coventry and Warwickshire NHS Trust, Coventry, GBR; 3 Emergency Medicine, Yorkshire Ambulance Service NHS Trust, West Yorkshire, GBR; 4 Morbid Anatomy, University of Nigeria Teaching Hospital, Enugu, NGA; 5 Internal Medicine, Babcock University Teaching Hospital, Ilishan-Remo, NGA; 6 Internal Medicine, Benjamin S. Carson College of Health and Medical Sciences, Ilishan-Remo, NGA; 7 Surgery, The Chiltern Hospital, Great Missenden, GBR; 8 Medicine, St. Francois Medical Centre, Abuja, NGA; 9 Family Medicine, Federal Medical Centre (FMC), Umuahia, Umuahia, NGA; 10 Pediatrics, Nottingham University Hospitals NHS Trust, Nottingham, GBR; 11 Oncology/Toxicology, Nigeria Police Directorate of Medical Services, Abuja, NGA; 12 Pharmacology and Therapeutics, Ebonyi State University, Abakaliki, NGA; 13 Internal Medicine, King Fahad Specialist Hospital, Tabuk, SAU; 14 Obstetrics and Gynecology, Babcock University Teaching Hospital, Ilishan-Remo, NGA; 15 Surgery, Babcock University Teaching Hospital, Ilishan-Remo, NGA

**Keywords:** alpha-feto protein, mature cystic teratoma, mixed germ cell tumors, postmenopausal, treatment, yolk sac tumor

## Abstract

Mixed germ cell tumours (GCTs) are a rare pathology, particularly those comprising yolk sac tumour (YST) and mature cystic teratomas. Although one important biomarker for YSTs is alpha-fetoprotein (AFP), which is often characteristically elevated, this may not be the case in a minority of cases. Additionally, AFP is not usually elevated in mixed GCTs, more so in cases involving mature cystic teratoma, even with YST as a component of the tumour, as seen in the index patient who had a normal AFB value of 5 ng/mL (normal: 0-10 ng/mL).

Furthermore, in postmenopausal women with stage 1 mixed GCTs, the preferred treatment is non-fertility-sparing surgery without chemotherapy. Despite the generally poor prognosis for GCTs in this demographic, early detection and treatment can lead to favourable outcomes.

This report details a case of a 58-year-old woman with a stage 1 mixed GCT who underwent extensive surgery without chemotherapy and has remained tumour-free for 24 months, attributing her good prognosis to the low stage and early intervention, along with the YST component.

## Introduction

Ovarian germ cell tumours (GCTs) originate from the primordial germ cells of the ovaries, which are the embryonic cell precursors that subsequently transform into ovaries [1]. These tumours include embryonal carcinoma, dysgerminoma, choriocarcinoma, yolk sac tumours (YSTs), immature cystic teratomas, and mixed GCTs [2,3].

GCTs represent 20-30% of all ovarian neoplasms, with 95% of GCTs benign and only 2-3% malignant [1,2,4]. GCTs are the most common ovarian tumour in the first three decades of life, with a peak incidence between ages 16 and 20 [4,5], with a good prognosis because most diagnoses are early and tumours are exquisitely sensitive to chemotherapy [6]. However, they are incredibly rare in women between the ages of 50 and 86 years. It is also noted that the occurrence of GCTs in these age groups is associated with moribund outcomes due to advances in age and possible malignant transformation [7].

Furthermore, mixed GCTs represent a relatively uncommon neoplasm composed of two or more germ cell components. Among mixed GCTs, dysgerminoma is the most frequently observed component, occurring in approximately 80% of cases, followed by YSTs (70%), immature teratomas (53%), choriocarcinomas (20%), and embryonal carcinomas (16%). According to the literature, the combination of dysgerminoma and YSTs is the most reported, accounting for approximately one-third of all mixed GCT cases [1,6]. A mature cystic teratoma and a YST occurring together represent an uncommon variant of ovarian mixed GCTs.

The clinical presentation of GCT varies; they are commonly noticed to have a sub-acute pattern of presentations because GCTs, unlike epithelial ovarian tumours, grow rapidly and present with a rapidly enlarging pelvic mass attributed to capsular distension, haemorrhage and necrosis, pressure symptoms on bowels and bladder, and menstrual irregularities in pre-menopausal patients [1].

Mixed GCTs can secrete either alpha-fetoprotein (AFP), beta-hCG, or a combination of both, depending on the components of the tumour. The primary course of treatment consists of surgery, primarily tumour removal, with adjuvant chemotherapy. Bleomycin, etoposide, and cisplatin (BEP) is the preferred chemotherapy combination in advanced or metastatic cases of mixed GCTs, recurrent diseases, high-grade tumours, and mixed GCTs with aggressive components such as YST, choriocarcinoma, or embryonal carcinoma. 

We herein discuss a rare case of a YST co-existing with a mature cystic teratoma in a 58-year-old postmenopausal woman.

## Case presentation

A 58-year-old woman, 10 years post-menopausal, presented to our gynaecology clinic with a two-month history of dull abdominal pain, rapidly growing abdominal mass, and constipation. Physical examination revealed a cachectic patient with severe pallor. The abdomen was asymmetrical and grossly distended, with periumbilical tenderness. A right-sided firm pelvic mass about the size of a 26-week pregnancy was palpated. Rectal examination was normal. An initial assessment of large bowel obstruction secondary to the cause was made.

On laboratory investigations done on the first day of presentation, tumour markers AFP, carcinoembryonic antigen (CEA), cancer antigen 125 (CA 125), and lactate dehydrogenase (LDH) were within the normal range: AFP = 5 ng/mL (normal = 0-10 ng/mL), CEA = 1 ng/mL (normal = 0 to 3 ng/mL), CA 125 = 12 (normal 0-19 U/mL), and LDH = 142 (normal = 135-214 U/L). These normal levels of AFP and LDH contrast with expected elevations in YST and mixed germ cells, where their elevations serve as important tumour markers. Also, other investigations, including clotting profile and renal function test, were within the normal range. However, the haemoglobin (Hb) concentration level was 7.0 g/dL, which agrees with the clinical examination finding of severe anaemia.

The CT scan done on the first day of presentation revealed a large, multiloculated cystic mass with attenuation values ranging from 2 to 16 HU. The mass originated from the left adnexa and extended towards the right pelvis and abdomen, with its epicentre located in the right lumbar region, a finding that agrees with the clinical abdominal examination. The mass had areas of fat density (148-153 HU) and calcifications (609-1056 HU). The mass was 21.9 × 18.0 × 18.89 cm in size and had a volume of about 3,872 mL (Figure 1). The overall features were suggestive of a left ovarian teratoma.

**Figure 1:**
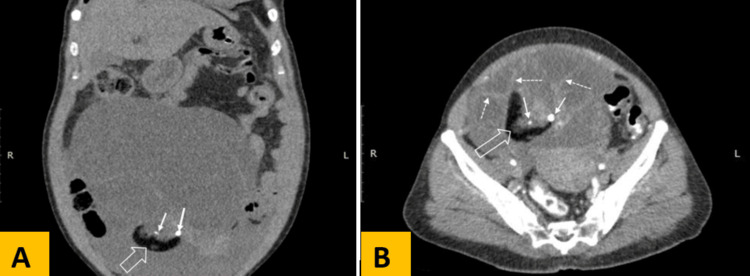
Non-contrast coronal reformatted (A) and contrast-enhanced axial (B) abdominopelvic computed tomography (CT) images showing a large multiseptated intra-abdominal cystic mass (broken white arrows) arising from the left adnexa. A hypodense area with fat attenuation (transparent arrow) and foci of calcification (solid white arrow) are also identified within the cyst. These imaging features are suggestive of a left ovarian teratoma.

She was transfused with two units of fresh whole blood on the second and third days of presentation, respectively.

During surgery done on the fourth day of presentation, a total abdominal hysterectomy with bilateral salpingo-oophorectomy and infracolic omentectomy was performed. Approximately 200 mL of haemorrhagic ascitic fluid was noted. The right ovary and fallopian tube were atrophic, while the left ovary was grossly enlarged, a finding that was supported by the presurgical CT scan evaluation. No obvious tumour was observed, and the remaining visceral organs appeared grossly normal.

Pathological gross examination of the left ovary done on the seventh day of presentation showed that it weighed 2.5 kg and measured 20.0 × 16.0 × 12.0 cm. Sectioning revealed multiple cystic cavities containing a chocolate-colored fluid, a golden-yellow fluid, gelatinous material, and tufts of hair. The walls of the cyst had solid areas that were not evenly distributed.

Microscopic analysis done on the 11th day of presentation revealed a germ cell neoplasm composed of two distinct components. The first component was consistent with a mature cystic teratoma (as was suggested by the presurgical CT scan), characterised by cystic lesions lined by epidermis. The cyst wall included adnexal structures, including sebaceous glands and hair follicles (Figures 2, 3).

**Figure 2:**
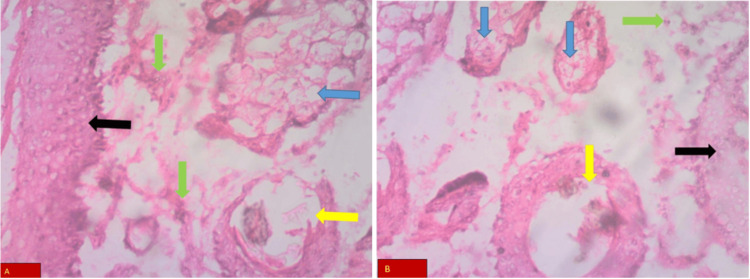
Haematoxylin and eosin (H&E) staining at ×100 magnification showing skin adnexa structures (blue and yellow arrows), epidermis (black arrow), and tumour cells. (A): H&E at x100 showing lobules of sebaceous glands (blue arrows), epidermis (black arrow), hair follicle (yellow arrow), and tumour cells disposed in a vague reticular and single-cell patterns (green arrows) in an oedematous-to-myxoid stroma. (B): H&E at x100 showing lobules of sebaceous glands (blue arrows), epidermis (black arrow), hair follicle (yellow arrow), and tumour cells disposed in a relatively more prominent reticular and single-cell patterns (green arrow). Overall features in both (A & B) are suggestive of a mature cystic teratoma co-existing with another tumour.

**Figure 3:**
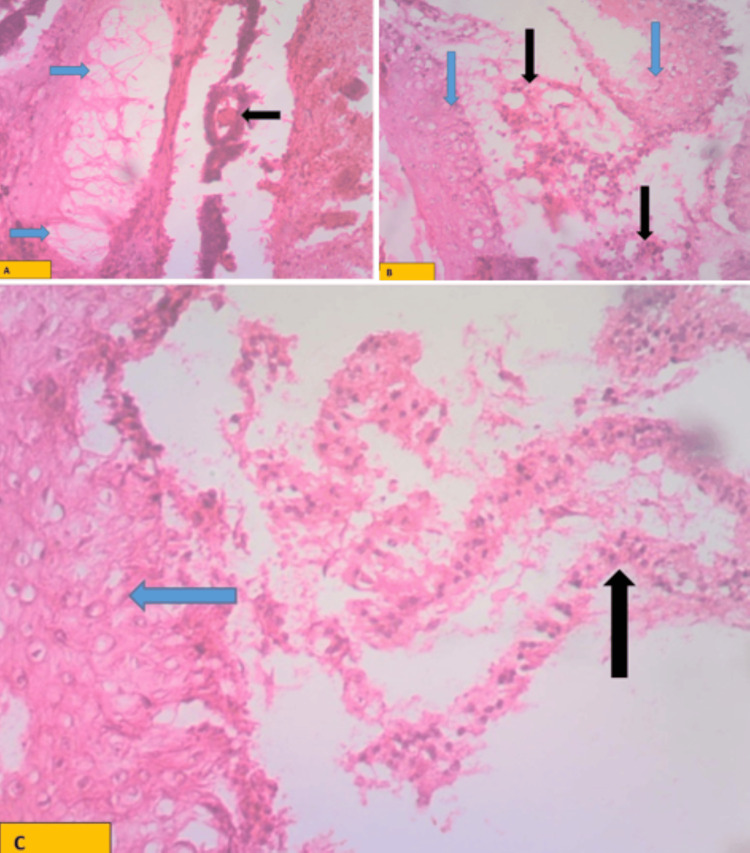
Haematoxylin and eosin (H&E) staining at ×100 magnification showing microscopic features of a mature cystic teratoma coexisting with a yolk sac tumour. (A): H&E at x100 showing lobules of mature adipocytes (blue arrows) and a nearby focal Schiller-Duval body (black arrow). (B): H&E at x100 showing epidermis (blue arrows) and tumour cells disposed in a reticular pattern (black arrows). (C) H&E at x100 showing epidermis (blue arrow) and tumour cells disposed in a festoon to a glandular pattern (black arrow). Overall features in (A, B, and C) are consistent with a mature cystic teratoma co-existing with a yolk sac tumour.

The second component corresponded to a YST, with tumour cells arranged in reticular, glandular, papillary, festoon, and mesenchymal-like patterns, including cords and tubules of tumour cells scattered within an oedematous-to-myxoid stroma (Figures 3, 4). The tumour cells exhibited variable cytological atypia, with hyperchromatic-to-vesicular nuclei, some with prominent nucleoli, and moderate pale eosinophilic-to-clear cytoplasm (Figures 4, 5).

**Figure 4:**
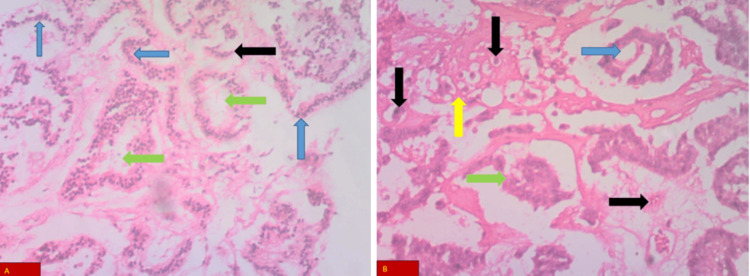
Haematoxylin and eosin (H&E) staining at ×100 showing tumour cells with microscopic features suggestive of a yolk sac tumour. (A): H&E at x100 showing tumour cells disposed in festoon (blue arrows), cord (black arrow), and gland-like (green arrows) patterns within an oedematous-to-myxoid stroma. (B): H&E at x100 showing tumour cells disposed in glandular (blue arrow), reticular (yellow arrow), and papillary (green) patterns. The tumour cells are atypical, having hyperchromatic-to-vesicular nuclei, some with prominent nucleoli, and moderate pale eosinophilic-to-clear cytoplasm (black arrows). Overall features in both (A & B) are suggestive of a yolk sac.

**Figure 5:**
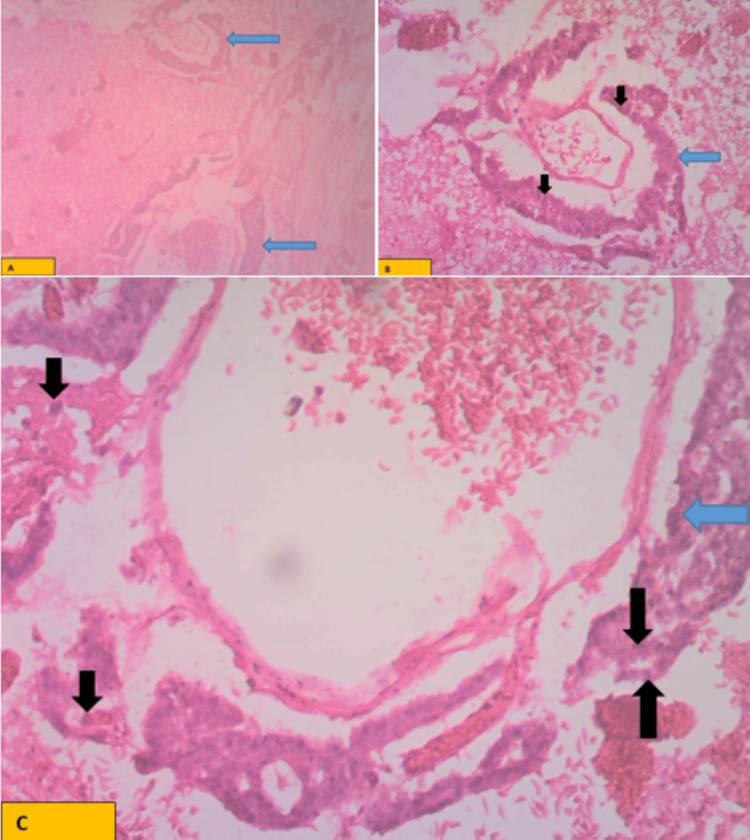
Haematoxylin and eosin (H&E) staining showing Schiller-Duval bodies (blue arrows). Low-power magnification at ×40 (A) and higher-power magnification at ×100 (B and C). (A): H&E at x40 (low magnification) showing Schiller-Duval bodies (blue arrows). (B): H&E at x100 (higher magnification) showing a Schiller-Duval body (blue arrow). (C): H&E at x100 (higher magnification) showing another Schiller-Duval body (blue arrow). The component tumour cells in (B & C) are atypical with hyperchromatic-to-vesicular nuclei, some with prominent nucleoli, and moderate pale eosinophilic-to-clear cytoplasm (black arrows). Overall features in (A, B and C) are consistent with a yolk sac tumour.

Focal areas of Schiller-Duval bodies, haemorrhage, and necrosis were also observed (Figures 3, 5). These findings are diagnostic of a mixed GCT, comprising a YST and a mature cystic teratoma.

## Discussion

Ovarian GCTs are derived from primordial germ cells of the ovaries [1]. According to the WHO, ovarian GCTs are classified into many histological subtypes, including dysgerminoma, YSTs, embryonal carcinoma, polyembryoma, choriocarcinoma, teratomas, and mixed GCTs [8]. Among children and adolescents, over half of ovarian tumours are germ cell in origin, with one-third being malignant. In contrast, GCTs are relatively uncommon in adults, where the majority are benign, predominantly mature cystic teratomas (dermoid cysts). Histologically, dysgerminomas are the prototypical GCTs, while embryonal carcinomas consist of poorly differentiated, multipotential germ cells. Germ cell differentiation along embryonal or somatic pathways leads to teratomas, while extraembryonic differentiation produces YSTs or choriocarcinomas. Mixed GCT subtypes are also infrequently observed [8].

GCTs represent 20-30% of all ovarian tumours, with only 2-3% of them being malignant [2,7]. Mixed GCTs contain two or more types of germ cells combined. The majority of ovarian mixed GCTs show a combination of two germ cell components; the most common components documented are dysgerminoma with YST, followed by YST and immature teratoma. It is also reported that tumours showing combinations of three germ cell components have an incidence of 14-31% and 2-10% for those composed of four germ cell components [9,10]. Our patient had an uncommon combination of GCTs comprising a mature cystic teratoma and a YST.

The clinical presentation of mixed GCTs is usually subacute and similar to that of other ovarian tumours. The subacute nature of the presentation is attributed to the rapid growth of ovarian GCTs, usually due to intracapsular haemorrhage, necrosis, and capsular distension. However, it is worthy of note that because of the massive size of the tumour, GCTs can present acutely due to tumour rupture, torsion and haemorrhage [1,6]. Common clinical presentations include gross abdominal distension from the voluminous ovarian mass, abdominal distension, abdominal pain, pressure symptoms, amenorrhoea, and abnormal uterine bleeding [9,10]. The index patient presented with abdominal pain, a rapidly growing abdominal mass, and pressure symptoms (constipation), which gave an erroneous impression of intestinal obstruction, consistent with the literature.

Depending on the histological subtype present in the mixed GCTs, they can secrete a combination of serum tumour markers, including AFP, beta-human chorionic gonadotropin, LDH, and placental alkaline phosphatase, and they are valuable tests for the diagnosis, management, and monitoring of these GCTs. Surprisingly, even with the diagnosis of YST as a component of our patient’s mixed GCT, AFP was within the normal range, as is usually the case in over 90% of cases in which it serves as an important marker of diagnosis [10]. However, this expected elevated AFP may not always occur in mixed GCTs, particularly when a teratoma is a component, even in the presence of YST [11].

Radiologically mixed GCTs appear on both MRIs and CTs as voluminous masses with heterogeneous compositions of solid and cystic components with or without areas of calcification, haemorrhage, or necrosis [12]. In our case, our patient's CT scan showed a large, multiloculated cystic mass with calcifications.

Treatment options usually depend on a myriad of factors: the patient's age, preservation of fertility, and probability of a malignant component. Fertility-sparing surgery, along with active surveillance and chemotherapy, is based on the histological subtype of the tumour, its staging, and tumour characteristics [3].

Fertility-sparing surgery is crucial for women under 40 with malignant ovarian GCTs, as studies show these procedures yield outcomes similar to radical surgeries when properly monitored. For early-stage GCTs, options like unilateral salpingo-oophorectomy are preferred, often not requiring adjuvant chemotherapy. Advances in chemotherapy have improved GCT outcomes, allowing for successful pregnancies post-surgery [3].

Conversely, non-fertility-sparing surgery is reserved for advanced cases or postmenopausal women, typically involving abdominal hysterectomy and bilateral salpingo-oophorectomy, coupled with comprehensive surgical staging. For patients with residual immature teratomas or growing teratoma syndrome following adjuvant chemotherapy, second-look surgeries may be considered to address incompletely resected tumour elements [3,13].

Overall, the decision between surgery types must balance oncological safety with reproductive goals and is influenced by tumour stage and patient circumstances. Both approaches demonstrate the potential for favourable outcomes with appropriate management [3]. Additionally, the recommended adjuvant chemotherapy for mixed GCT includes BEP administered over four to six cycles [14], while salvage regimens alongside secondary cytoreductive procedures address resistant tumours [7]. Our patient had a more radical approach to her surgery, as she is postmenopausal and had completed her family size; also, adjuvant chemotherapy was not given because intraoperative staging revealed a stage 1 tumour.

Prognostic factors for mixed GCTs include primary lesion size, early diagnosis, age, chemotherapy initiation, and the malignant component's proportion. Surgical stage IA mixed GCTs under 10 cm have a 100% survival rate. Mixed GCTs with YST, choriocarcinoma, or grade 3 immature teratoma as components, when these malignant elements are less than one-third of the tumour, show an excellent prognosis [3,6]. Despite generally poor prognoses in postmenopausal women and her huge tumour size of a 26-week pregnancy (35 cm), our patient has remained tumour-free for 24 months due to low tumour stage and early diagnosis. The YST, as a component of the mixed GCT, in her case, may have also contributed to her favourable outcome.

## Conclusions

In our case, we did not observe an increase in AFP, which is a rare occurrence in YST. Even with YST as a component of the tumour, AFP is typically not increased in cases of mixed GCTs involving mature cystic teratomas. Postmenopausal women with stage 1 illness typically undergo total abdominal hysterectomy with bilateral salpingo-oophorectomy, excluding chemotherapy. Even though GCTs are known to have a negative outlook in postmenopausal women and after the third decade of life, favourable outcomes can be achieved with low-stage tumours and early detection and treatment at presentation. Furthermore, a comparatively better prognosis may arise in GCTs that include YST as a component.
